# Decrease of Urinary Nerve Growth Factor but Not Brain-Derived Neurotrophic Factor in Patients with Interstitial Cystitis/Bladder Pain Syndrome Treated with Hyaluronic Acid

**DOI:** 10.1371/journal.pone.0091609

**Published:** 2014-03-10

**Authors:** Yuan-Hong Jiang, Hsin-Tzu Liu, Hann-Chorng Kuo

**Affiliations:** 1 Department of Urology, Buddhist Tzu Chi General Hospital and Tzu Chi University, Hualien, Taiwan; 2 Institute of Pharmacology and Toxicology, Tzu Chi University, Hualien, Taiwan; National Cancer Institute at Frederick, United States of America

## Abstract

**Aims:**

To investigate urinary nerve growth factor (NGF) and brain-derived neurotrophic factor (BDNF) levels in interstitial cystitis/bladder pain syndrome (IC/BPS) patients after hyaluronic acid (HA) therapy.

**Methods:**

Thirty-three patients with IC/BPS were prospectively studied; a group of 45 age-matched healthy subjects served as controls. All IC/BPS patients received nine intravesical HA instillations during the 6-month treatment regimen. Urine samples were collected for measuring urinary NGF and BDNF levels at baseline and 2 weeks after the last HA treatment. The clinical parameters including visual analog scale (VAS) of pain, daily frequency nocturia episodes, functional bladder capacity (FBC) and global response assessment (GRA) were recorded. Urinary NGF and BDNF levels were compared between IC/BPS patients and controls at baseline and after HA treatment.

**Results:**

Urinary NGF, NGF/Cr, BDNF, and BDNF/Cr levels were significantly higher in IC/BPS patients compared to controls. Both NGF and NGF/Cr levels significantly decreased after HA treatment. Urinary NGF and NGF/Cr levels significantly decreased in the responders with a VAS pain reduction by 2 (both p < 0.05) and the GRA improved by 2 (both p < 0.05), but not in non-responders. Urinary BDNF and BDNF/Cr did not decrease in responders or non-responders after HA therapy.

**Conclusions:**

Urinary NGF, but not BDNF, levels decreased significantly after HA therapy; both of these factors remained higher than in controls even after HA treatment. HA had a beneficial effect on IC/BPS, but it was limited. The reduction of urinary NGF levels was significant in responders, with a reduction of pain and improved GRA.

## Introduction

Interstitial cystitis/bladder pain syndrome (IC/BPS) is a chronic inflammatory disorder of the urinary bladder characterized by bladder pain associated with frequency, urgency, nocturia, and sterile urine. The aetiology of IC/BPS remains unclear and is thought to be multifactorial, including defective or dysfunctional bladder urothelium, neurogenic inflammation, activation of mast cells, autoimmunity, and occult infection [1], [2], [3]. In IC/BPS, disruption of the urothelial barrier initiates a cascade of events in the bladder, leading to symptoms and disease [4]. Specifically, epithelial dysfunction leads to the migration of urinary solutes, in particular, potassium that depolarizes nerves and muscles and causes tissue injury. Suburothelial and neurogenic inflammation in IC/BPS inhibits normal basal cell proliferation and affects apical urothelial function, forming a vicious cycle provoking and maintaining inflammatory reactions in the bladder [1], [5].

Glycosaminoglycan (GAG) is a part of the normal bladder epithelium as a chemical barrier and protects the bladder mucosa from bacterial adhesion and penetration by toxic substances in the urine [6]. In IC/BPS patients, this GAG layer is thought to be partially defective, causing a defect in the permeability barrier of the urothelium [7]. Infiltrated urine would induce submucosal inflammation, stimulating sensory nerve fibers and causing frequency and pain [8], although the reason why the GAG layer becomes defective has been unknown. In addition, the urinary GAG profile and hyaluronic acid (HA) levels significantly correlated with IC/BPS severity [9]. A few uncontrolled or non-interventional studies of intravesical HA treatment of IC/BPS patients showed symptomatic improvement over a broad range between 30% and 85% of patients [10], [11], [12], [13], [14], [15], [16]. However, these studies lacked solid evidence from multi-centre, randomized controlled trials to show the long-term superiority and the anti-inflammatory effect of intravesical HA treatment for IC/BPS.

Nerve growth factor (NGF) and brain-derived neurotrophic factor (BDNF) are the most studied neurotrophins in the bladder, governing innervation during development, growth, and injury [17], [18]. NGF and BDNF are expressed in the urothelium and bladder smooth muscle, influencing lower urinary tract symptoms of overactive bladder (OAB) and IC/BPS [17], [18], [19]. Urinary neurotrophins can be used as biomarkers to improve the accuracy of diagnosing OAB and IC/BPS and to monitor the effectiveness of treatment [19], [20].

Previous study has shown increased urinary chemokines and cytokines (including NGF) had different expression among control, ulcerative and non-ulcerative IC/BPS [21]. In our previous study, increased serum and urinary NGF levels in IC/BPS suggest that chronic inflammation is involved in this bladder disorder [22]. This study was designed to investigate the changes of urinary NGF and BDNF in IC/PBS patients after intravesical HA treatment, and to identify objective and reliable biomarkers of IC/BPS.

## Materials and Methods

This prospective study was conducted from January 2009 to December 2010 in a tertiary teaching hospital. A total of 33 consecutive patients with IC/BPS were enrolled. IC/BPS was diagnosed following the East Asian guidelines [1] and was based on the characteristic symptoms of suprapubic pain accompanied by urinary frequency, nocturia and cystoscopic findings of glomerulation. The patients with Hunner’s lesions in cystoscopy were excluded, and all of the eligible patients in this study were non-ulerative IC/BPS. All patients with characteristics of IC/BPS were previously treated conservatively with heparin and/or pentosan polysulfate and were refractory to treatment. A total of 45 people without urological disease or lower urinary tract symptoms were recruited from patients and hospital employees and served as the control subjects.

All IC/BPS patients received nine times of intravesical HA instillations (50ml, 0.08% sodium hyaluronate) (Bioniche, Canada) over the course of a 6-month period, including four weekly intravesical instillations of 40 mg of HA followed by 5 monthly HA instillations. Urine samples were collected for measuring urinary NGF and BDNF levels at baseline (immediately before the first treatment) and 2 weeks after the last HA treatment.

Clinically, patients were assessed for pain using 10-point visual analogue scale (VAS). Patients were requested to measure their bladder pain elicited at full bladder from 0 (no pain at all) to 10 (extremely painful) at baseline and after treatment. In addition, a three-day voiding diary to record daily frequency and nocturia episodes, and functional bladder capacity (FBC, the maximum volume voided in the voiding diary) were also recorded. The treatment outcome was also assessed using the global response assessment (GRA) [23], [24]. Patients were requested to rate their bladder symptoms compared to baseline on a 7-point centered scale from markedly (−3), moderately (−2) and slightly worse (−1), no change (0), to slightly improved (+1), moderately improved (+2), and markedly improved (+3). Patients with moderately and markedly improved results after treatment (≥2) were considered to have a successful treatment outcome. The baseline urinary NGF and BDNF levels were compared between the controls and IC/PBS patients. In addition, after HA treatment, the urinary NGF and BDNF levels in IC/BPS patients were compared with the corresponding baseline data.

The present study was approved by the institutional Review Board and Ethics Committee of Buddhist Tzu Chi General Hospital. Each patient was informed about the study rationale and procedures, and written informed consent was obtained before the treatment.

### Urinary NGF and BDNF Measurement

Urinary NGF and BDNF levels were measured using the enzyme-linked immunosorbent assay (ELISA) method [25]. Voided urine was put on ice immediately and transferred to the laboratory for preparation. The samples were centrifuged at 3,000 rpm for 10 minutes at 4°C. The supernatant was separated into aliquots in 1.5-ml tubes and preserved in a freezer at −80°C. At the same time, 3 mL of urine was taken to measure the urinary creatinine level.

The NGF and BDNF concentrations were determined using the Emax ImmunoAssay System (Promega, Madison, WI, USA), with a precise and highly sensitive ELISA kit, which had a minimum sensitivity of 7.8 pg/ml. Assays were performed according to the manufacturer’s instructions. Briefly, NGF and BDNF levels were determined using an antibody sandwich format in 96-well plates. Each well was initially coated with 100 µL of anti-NGF/BDNF polyclonal antibody and incubated overnight at 4°C, followed by 1-h incubation with blocking buffer to prevent nonspecific binding. Either 100 ml of urine or 100 µl of NGF/BDNF standard (0–250 pg/mL) was added to each well followed by incubation for 6 hours at room temperature with shaking. Then, the plate was washed, anti-NGF/BDNF monoclonal antibody was added, and the plate was incubated at 4°C for 14–18 hours. After the plate had been washed again, the amount of bound monoclonal antibody was detected using IgG-horseradish peroxidase-conjugated antibody as a tertiary reactant. The unbound conjugate was removed by washing, and the plate was then incubated with 100 µL of TMB (3,3'5,5' tetramethyl benzydine) substrate solution for 10 minutes at room temperature. Hydrochloric acid (1 N, 100 µL) was added to terminate the reactions. The colour change was measured with a Synergy HT Microplate Reader (Bio-Tek Instruments) at 450 nm. The amounts of NGF and BDNF in each urine sample were determined from a standard curve. All samples were run in triplicate, and the values were averaged. Total urinary NGF and BDNF levels were normalized by urinary creatinine concentration (NGF/Cr and BDNF/Cr levels). ELISA assay for urinary NGF and BDNF was performed at baseline, and after completed HA treatment.

### Statistical Analysis

Normalized total urinary NGF/Cr and BDNF/Cr levels were expressed as means ± standard deviations. Unpaired Student’s *t* and Mann-Whitney tests were used for statistical analysis between groups when considering parametric and nonparametric data, respectively. Pearson correlation was used to evaluate the associations between the variations in urinary NGF/Cr and BDNF/Cr levels. A *P* value < 0.05 was considered to indicate statistical significance; all tests were two-tailed. All statistical analyses were performed with the statistical package SPSS for Windows (Version 12, SPSS, Chicago, IL, USA)

## Results

The mean age was 46.9±14.0 years (range, 16–61 years) among the 33 IC/BPS patients and 45.9±14.0 years (range, 19–58 years) among the 45 controls (p  =  0.77). There were five male patients each among the IC/BPS patients and controls.

At baseline, urinary NGF, NGF/Cr, BDNF and BDNF/Cr levels were significantly higher in IC/BPS patients compared to the controls (Table 1). After the 6-month intravesical HA treatment regimen for IC/BPS patients, urinary NGF and NGF/Cr levels both decreased significantly while urinary BDNF and BDNF/Cr levels did not. In addition, clinical assessments including VAS, GRA and FBC all significantly improved after treatment. Although the decrease of urinary NGF and NGF/Cr levels and the improvement of clinical parameters were noted after HA treatment, urinary NGF, NGF/Cr and BDNF levels in HA treated IC/BPS patients were still significantly higher than in controls, except for urinary BDNF/Cr levels (p  =  0.08).

**Table 1:** Comparison of the clinical characteristics and urinary nerve growth factor (NGF), NGF/creatinine (NGF/Cr), brain-derived neurotrophic factor (BDNF), and BDNF/Cr levels between controls and IC/BPS patients.

	Controls (N = 45)	Baseline IC/BPS patients (N = 33)	Post-HA treatment IC/BPS patients (N = 33)	p value*	p value#	p value$
**Urinary NGF (pg/ml)**	2.25±0.91	92.4±24.9	34.2±10.6	0.04	<0.01	<0.01
**Urinary NGF/Cr (pg/mg)**	0.37±0.13	31.1±12.0	8.13±2.81	0.02	<0.01	< 0.01
**Urinary BDNF (pg/ml)**	9.57±5.37	107.5±40.8	64.1±21.0	0.68	<0.01	<0.01
**Urinary BDNF/Cr s(pg/mg)**	4.82±2.45	23.8±7.70	17.2±7.37	0.55	<0.01	0.08
**VAS**		3.12±2.47	1.79±2.07	<0.01		
**GRA**		1.09±0.84	1.85±0.94	<0.01		
**FBC (ml)**		171±92.5	229±118	<0.01		

*: p value between baseline and post-HA treatment data in IC/BPS patients.

# : p value between the baseline data in controls and IC/BPS patients.

$ : p value between the data in controls and the post-HA treatment data in IC/BPS patients.

VAS: visual analogue score, GRA: global response assessment, FBC: functional bladder capacity.

Data are expressed as mean ± standard deviation.

**Figure 1:**
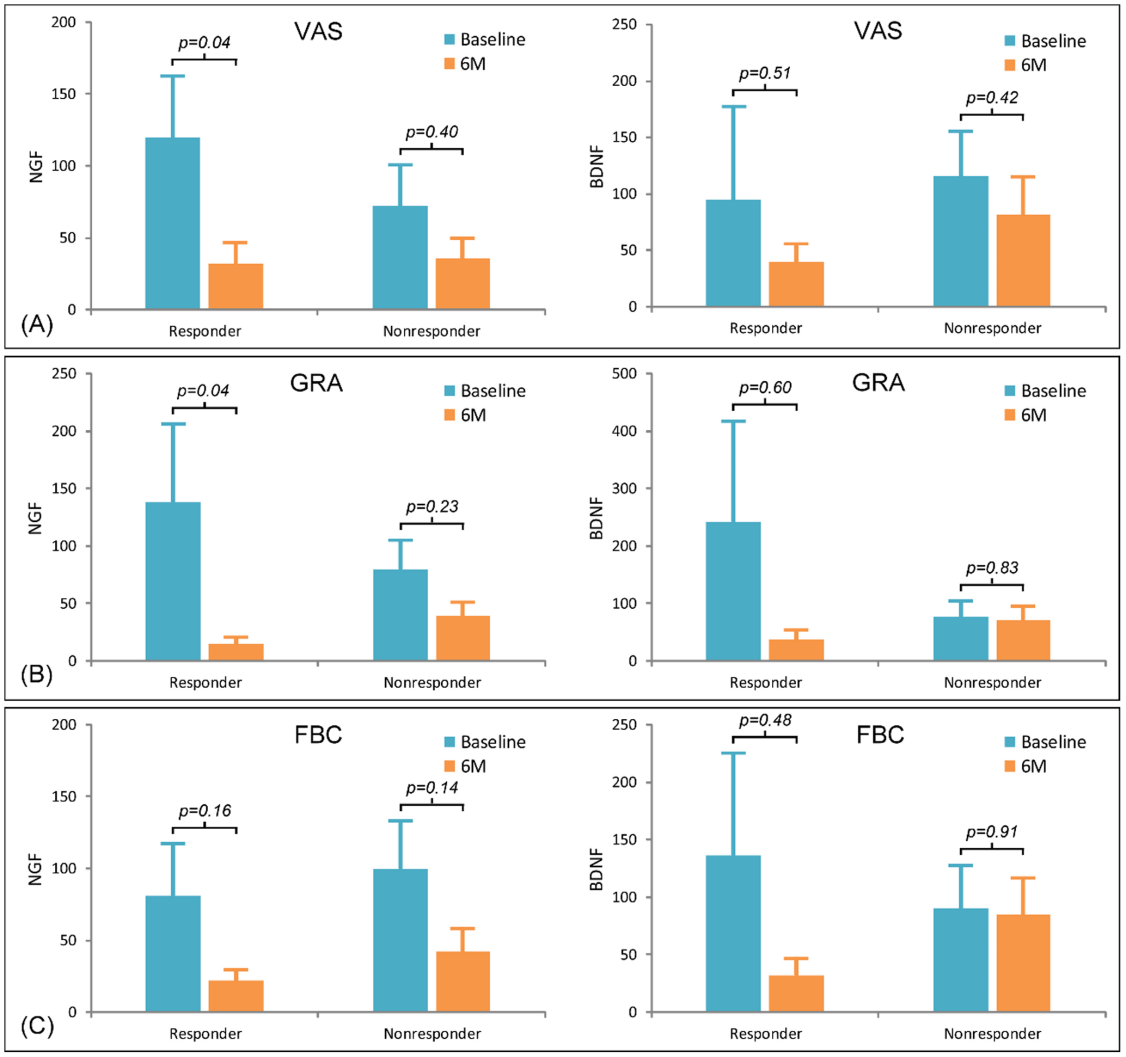
The changes of urinary levels of nerve growth factor (NGF) and brain-derived neurotrophic factor (BDNF). The urinary levels of NGF (pg/ml) and BDNF (pg/ml) were compared between baseline and after 6-month treatment of HA (6M) in responders and non-responders based on (A) a visual analogue scale (VAS, for pain) decrease of ≥ 2, (B) global response assessment (GRA) increase of ≥ 2 and (C) functional bladder capacity (FBC) increase of ≥ 50%. Data are expressed as means ± standard deviations.

After intravesical HA treatment, among the 33 IC/BPS patients, 14 patients (42.4%) experienced a pain VAS reduction of ≥ 2 (pain responders), 7 patients (21.2%) experienced a GRA reduction of ≥ 2 (GRA responders), and 13 patients (39.4%) experienced an FBC increase of ≥ 50% (FBC responders) (Table 2). Urinary NGF and NGF/Cr levels were significantly decreased in pain responders and in GRA responders, but not in non-responders (Table 2 and Fig. 1). However, there was no significant change in urinary NGF/Cr level, in either FBC responders or non-responders. Within either pain, GRA, and FBC responders or non-responders, there was no significant change of urinary BDNF or BDNF/Cr level after intravesical HA treatment.

**Table 2:** Comparison of urinary NGF/Cr and BDNF/Cr levels in clinical responders and non-responders after intravesical hyaluronic acid treatment in IC/BPS patients.

	Urinary NGF/Cr (pg/mg)			Urinary BDNF/Cr (pg/mg)		
	Baseline	HA treatment	P value	Baseline	HA treatment	P value
**VAS**						
Responder * (n = 14)	34.1±14.5	9.41±5.21	0.03	14.9±10.5	9.96±3.39	0.58
Non-responder (n = 19)	28.9±18.2	7.18±3.15	0.21	29.8±10.8	22.6±12.6	0.33
P value	0.52	0.73		0.12	0.70	
**GRA**						
Responder # (n = 7)	45.2±25.7	4.53±2.15	0.04	28.9±22.2	10.7±5.04	0.92
Non-responder (n = 26)	27.3±13.7	9.10±3.52	0.13	22.6±8.21	19.0±9.28	0.55
P value	0.40	0.79		0.68	0.38	
**FBC**						
Responder $ (n = 13)	25.7±14.3	4.34±1.23	0.14	24.0±11.5	6.76±2.35	0.29
Non-responder (n = 20)	34.6±17.7	10.6±4.54	0.06	23.6±10.4	24.0±11.9	0.98
P value	0.87	0.91		0.78	0.71	

* Responder in VAS: decrease of ≥ 2 scales.

# Responder in GRA: increase of ≥ 2 scales.

$ Responder in FBC: increase of ≥ 50% of baseline.

Data are expressed as mean ± standard deviation.

## Discussion

Urinary NGF and BDNF levels in IC/BPS patients were significantly higher than in the control subjects [22]. In IC/BPS patients after intravesical HA treatment, urinary NGF levels and clinical assessments including the pain VAS, GRA and FBC all significantly decreased compared to baseline, suggesting that HA treatment has an anti-inflammatory effect. However, the urinary NGF or BDNF levels after HA treatment were still significantly higher than in the controls, suggesting chronic inflammation in IC/BPS bladders could not be totally eradicated after one 6-month treatment course of HA.

HA is considered a good candidate for GAG layer substitution to provide a protective barrier for impaired urothelium in IC/BPS patients. HA prevents direct exposure of the urothelium to urine, bacterial adherence and the possibility of infection [26]. The restoration of urothelial barrier function should help prevent the vicious cycle of suburothelial inflammation and relieve bladder pain symptoms in IC/BPS patients.

NGF and BDNF are the most investigated neurotrophins in the lower urinary tract, especially in the bladder; they represent feasible diagnostic and treatment targets to improve bladder function [17], [18]. Elevated levels of circulating NGF might also increase the excitability or susceptibility of sensory receptors in suburothelial nerve fibers such as purinergic receptors P2X3 and transient receptors potential vanilloid receptor subfamily 1 (TRPV1) through intrinsic enhancement, causing the bladder to become more excitable, which in turn leads to symptoms of IC/BPS [27]. Endogenous NGF seems to contribute to lower urinary tract dysfunction through regulation of neural function, as well as inflammation and pain [25]. NGF applied in the bladder results in changes of bladder function, including bladder hyperreflexia, while affecting the visceral pain sensation by inducing hyperalgesia. It is proposed that these effects resemble pathologies associated with bladder overactivity and inflammatory pain, such as OAB and IC/BPS [18]. BDNF is the most abundant neurotrophin although less investigated, and little is known about the role of BDNF in bladder function both in normal and pathological conditions [17], [28]. Following chronic bladder inflammation and spinal cord injury, the synthesis of BDNF in the bladder is strongly increased [25], [29]. In addition, BDNF sequestration improved bladder function in rats with chronic cystitis [29].

In IC/BPS patients, NGF levels in both urine and serum increased, suggesting chronic inflammation is involved in the pathogenesis of the disease [22]. After cystoscopic hydrodistention treatment, a decrease in urinary NGF level was associated with greater pain reduction and a successful treatment response in IC/BPS patients [30]. Additionally, trigonal injection of botulinum toxin A improves IC/BPS symptoms, and significantly reduces urinary NGF and BDNF levels, even though the reduction is transient [31]. In our study, the reduction of urinary NGF level was significant in pain responders (decreased pain) and in those with GRA improvement. Altogether, both NGF and BDNF in the urine reflect the inflammatory conditions in patients with IC/BPS and indicate the clinical therapeutic effects of treatment.

In a rat model of cyclophosphamide-induced cystitis, HA was an effective treatment for bladder overactivity through the involvement of NGF signalling [32]. In this study, urinary NGF, but not BDNF, levels in IC/BPS patients decreased after intravesical HA treatment. However, urinary NGF, NGF/Cr, and BDNF levels in these HA treated patients were still significantly higher than in control subjects. Urinary NGF and NGF/Cr levels decreased significantly in pain and GRA responders. Interestingly, we did not find a significant decrease of urinary BDNF or BDNF/Cr in such responders or non-responders after HA therapy. There are two possible explanations for this phenomenon. The first is that HA had a limited anti-inflammatory effect on IC/BPS, causing significant decreases in only urinary NGF and NGF/Cr, but not BDNF or BDNF/Cr (although the levels decreased, the reduction was not statistically significant). Further management including repeated intravesical HA instillation or intravesical botulinum toxin A injection is necessary to eradicate the chronic suburothelial inflammation of IC/BPS. Additionally, all the percentage of the responders in VAS, GRA, or FBC did not exceed 50% after HA treatment. Secondly, changes in these two neurotrophin levels after HA treatment might reflect their different roles of chronic inflammation in IC/BPS.

In a previous prospective randomized study, two different HA regimens to treat IC/BPS provided similar significant improvements in symptoms scores and quality of life index during the 6-month treatment period [33]. Nonetheless, there is remarkably little objective laboratory evidence to indicate the therapeutic effects of HA on IC/BPS. The present study shows a significant change in urinary NGF level after HA treatment, which not only shows the therapeutic role of HA in the treatment of IC/BPS, but also indicates that urinary NGF could be considered a potential biomarker to evaluate the clinical response to IC/BPS treatment.

This study has some limitations. Although this was a prospective study, the sample size was small and from a single centre. In addition, since there was no placebo arm in this study, the effect of intravesical placebo instillation in these IC/BPS patients could not be determined. The collection of urine samples for measuring neurotrophic factors was not standardized to voided urine volume. Both the room temperature and the time from sample collection to laboratory might affect the protein measurements. More specific and standardized procedures for the collection of urinary neurotrophic factors are required in the future work.

## Conclusion

Urinary NGF, but not BDNF, levels decreased significantly after intravesical HA therapy for IC/BPS, but these two factors in HA treated patients were still higher than in the control subjects. The therapeutic effects of HA on IC/BPS manifested but were limited. The reduction of urinary NGF level was significant in pain responders (decreased pain) and in those with GRA improvement.
